# Biologically-inspired adaptive obstacle negotiation behavior of hexapod robots

**DOI:** 10.3389/fnbot.2014.00003

**Published:** 2014-01-29

**Authors:** Dennis Goldschmidt, Florentin Wörgötter, Poramate Manoonpong

**Affiliations:** ^1^Bernstein Center for Computational Neuroscience, Third Institute of Physics, Georg-August-Universität GöttingenGöttingen, Germany; ^2^Institute of Neuroinformatics, University of Zurich and ETH ZurichZurich, Switzerland; ^3^Mærsk Mc-Kinney Møller Institute, University of Southern DenmarkOdense, Denmark

**Keywords:** obstacle negotiation, autonomous robots, neural control, adaptive behavior, associative learning, backbone joint control

## Abstract

Neurobiological studies have shown that insects are able to adapt leg movements and posture for obstacle negotiation in changing environments. Moreover, the distance to an obstacle where an insect begins to climb is found to be a major parameter for successful obstacle negotiation. Inspired by these findings, we present an adaptive neural control mechanism for obstacle negotiation behavior in hexapod robots. It combines locomotion control, backbone joint control, local leg reflexes, and neural learning. While the first three components generate locomotion including walking and climbing, the neural learning mechanism allows the robot to adapt its behavior for obstacle negotiation with respect to changing conditions, e.g., variable obstacle heights and different walking gaits. By successfully learning the association of an early, predictive signal (conditioned stimulus, CS) and a late, reflex signal (unconditioned stimulus, UCS), both provided by ultrasonic sensors at the front of the robot, the robot can autonomously find an appropriate distance from an obstacle to initiate climbing. The adaptive neural control was developed and tested first on a physical robot simulation, and was then successfully transferred to a real hexapod robot, called AMOS II. The results show that the robot can efficiently negotiate obstacles with a height up to 85% of the robot's leg length in simulation and 75% in a real environment.

## 1. Introduction

Insects are truly remarkable creatures with fascinating capabilities. Although their brains are much simpler structures than the human brain, this does not limit their capacity of generating a vast variety of complex behaviors (Chittka and Niven, 2009). In particular, insects exhibit tremendously agile and versatile locomotion. Furthermore, insects show the outstanding ability to adaptively move through their natural habitats (Ritzmann and Büschges, 2007). The adaptability of motor behavior in insects enables an effective response to unknown and novel situations. These observations have encouraged many roboticists to develop biologically-inspired robots and control schemes. Most of them aim to design robots emulating biomechanical properties of certain animals, including stick insects (Schneider et al., 2012), cockroaches (Cham et al., 2002; Kingsley et al., 2006), and ants (Lewinger et al., 2006). Others extract basic principles from biological systems to reduce system complexity by using hybrid leg-wheeled robots (Saranli et al., 2001; Lewinger et al., 2005). Several works use an inverse parametric model to control leg movements and posture of robots (Fielding et al., 2001; Gassmann et al., 2001), some of them take inspiration from biological control paradigms like Central Pattern Generators (CPGs) and local leg reflex controls (Espenschied et al., 1996; Klaassen et al., 2002). Only a few works have focused on obstacle negotiation. Most of them have increased climbing performance by using certain biomechanical improvements, such as a controllable body joint (Lewinger et al., 2005) and a “*sprawled posture*” (Pavone et al., 2006). Recent works on obstacle negotiation also have been done on hybrid leg-wheeled robots (Chen et al., 2011; Chou et al., 2011). These robots have fewer degrees of freedom and therefore can be more easily controlled than legged robots. However, in contrast to legged robots, these robots have problems performing versatile locomotion such as omnidirectional walking and negotiating very tall or narrow steps due to their reduced mobility.

Comparing the capabilities of current autonomous mobile robots with the agility of insects, one can easily notice the superiority of biological systems. Insects, such as cockroaches and stick insects, are able to adaptively negotiate very high obstacles compared to their body scale. Adaptive motor behavior enables insects not only to initiate different higher-order behaviors, such as climbing or turning, but also to locally adapt their leg and postural control (Ritzmann and Büschges, 2007; Schütz and Dürr, 2011). Recent insect studies have shown the use of antennae in gauging the distance from an obstacle to adapt leg and postural movements to successfully surmount an obstacle (Harley et al., 2009; Schütz and Dürr, 2011). The other components suggested by behavioral neurobiology are reactive key behaviors [i.e., a positive change in body-substrate angle, body flexion, center of mass (CoM) elevation, and local leg reflexes] of insect climbing (Fischer et al., 2001; Watson et al., 2002; Ritzmann et al., 2004). Some parts of these key behaviors have been utilized by previous studies (Klaassen et al., 2002; Lewinger et al., 2005; Pavone et al., 2006), though only few groups have applied adaptive mechanisms (Pavone et al., 2006) and machine learning algorithms (Lee et al., 2006) for obstacle negotiation. Adaptive mechanisms allow the robot to deal with unknown situations, making it possible to negotiate a wide variety of obstacles autonomously.

These findings have motivated us to develop an adaptive neural control mechanism for obstacle negotiation behavior of hexapod robots consisting of locomotion control, reactive control, and associative learning based on a distributed scheme (Verschure et al., 1992; Verschure and Voegtlin, 1998). The CPG-based locomotion control generates versatile and efficient walking behavior with a multitude of gaits while reactive controls utilizing all mentioned key behaviors of insect climbing is for leg reflexes and body posture control. The neural learning mechanism enables the robot to adapt its step climbing behavior according to changing conditions, i.e., different obstacle heights and walking patterns. As a result, the robot successfully learns the association of an early, predictive signal (conditioned stimulus, CS) and a late, reflex signal (unconditioned stimulus, UCS), both provided by ultrasonic sensors at the front of the robot. Consequently, after learning the robot finds an appropriate distance from the obstacle to initiate negotiating it. In the results, we will show that the neural learning mechanism leads to stable, adaptive behavior allowing the robot to overcome very high obstacles.

The following section describes the biological background used as inspiration for the design of an adaptive obstacle negotiation control for hexapod robots. In section 3, we present method and materials used in this study. First, we introduce the hexapod robot AMOS II and the robot simulation framework *LpzRobots* as the development and testing platforms of our proposed control system. The implementation of the neural control mechanism for adaptive obstacle negotiation of hexapod robots is described. Section 4 presents experimental results of the learning mechanism and the resulting obstacle negotiation behavior of AMOS II. Finally, in section 5, we discuss our results and provide an outlook of conceivable future work.

## 2. Biological background

Insects, such as cockroaches and stick insects, can easily deal with various kinds of obstacles on their way. This is due to the neural control and biomechanics of the animal that generate and coordinate adaptive and reactive behaviors necessary for efficient obstacle negotiation. Efficiency and success in surmounting obstacles are strongly reliant on sensory data gathered from the environment and the adaptability of motor behavior. It has been shown that the antennae are mainly involved in evaluating an obstacle and detecting its spatial parameters (i.e., distance and height) through various mechanosensors (Harley et al., 2009; Schütz and Dürr, 2011). These parameters have shown to be crucial in efficient obstacle negotiation in insects, especially as an indicator of when to initiate climbing (Watson et al., 2002). Varying these parameters leads to behavioral adaptations of insects.

In general, biological systems are able to adapt through learning (Staddon, 1983). It allows organisms to alter their behavior in order to succeed in unpredictable environments. Classical conditioning (Pavlov, 1927), a subclass of associative learning which has been found in a variety of different insects (Alloway, 1972), is applied here for adaptive, insect-like obstacle negotiation behavior. In classical conditioning, an animal learns to associate a previously neutral stimulus (conditioned stimulus, CS) and an unconditioned stimulus (UCS) when both are presented temporally paired. Before learning, the UCS elicits a behavioral response that is referred to as unconditioned response (UCR). After the conditioning procedure, the presence of only the CS is capable to cause a response, called the conditioned response (CR). Note that due to temporal causality the CS has to precede the UCS; thereby, the CS becomes a predictive stimulus of the UCS. Different associative learning mechanisms have been previously developed and applied to robots for generating adaptive behavior [(Grossberg and Schmajuk, 1989; Baloch and Waxman, 1991; Verschure et al., 1992; Verschure and Voegtlin, 1998; Wörgötter, 2003), see the Discussion section for details]. For our adaptive climbing control described in section 3, we apply input correlation (ICO) learning (Porr and Wörgötter, 2006) as a model for classical conditioning to associate distal and proximal distance signals provided by ultrasonic sensors of the robot. As a result, the robot can learn to find an optimal distance from an obstacle to initiate climbing.

Several neuroethological studies have identified at least four behaviors necessary for efficient obstacle negotiation in insects: a positive change in body angle, center of mass elevation, body flexion, and local leg reflexes. (Watson et al., 2002) examined neural control and kinematics in obstacle negotiation of the deathhead cockroach (*Blaberus discoidalis*). In preparation for climbing, a cockroach extends its front and middle legs leading to a positive change of the body-substrate angle which is defined as the angle between ground and torso axis. Significant alternations from normal walking behavior are not required for obstacles smaller than the height of their front leg swing trajectory. For higher obstacles the transitional phase from walking to climbing is anticipated before the front legs reach the obstacle. This anticipatory upward movement of the torso is called the rearing stage (Watson et al., 2002). Stick insects prepare for obstacle negotiation by tilting the prothorax upwards before placing their front leg on top of the obstacle (Schütz and Dürr, 2011). The tilting behavior also assures a positive change in body angle. Similar changes of the body angle have been observed in potato beetles (Pelletier and McLeod, 1994). After the tarsus touches the top of the obstacle, the coxal-trochanteral (CTr) joint extends to elevate the body lifting the center of mass (CoM) upwards. A translation of the CoM above the obstacle is required to enable insects to fully climb the obstacle. This is done by a stronger leg extension of the middle leg pair. This stage has been named the rising stage (Watson et al., 2002). In cockroach climbing, it appears that climbing does not require any remarkable dispositions of general walking control mechanisms, but manipulative alternations of posture and leg movements in the anticipatory behavior of the rearing stage. Besides kinematic stability, insects compensate perturbations during locomotion dynamically (Ghigliazza and Holmes, 2005; Spence et al., 2010) which requires a large set of sensory organs for force/torque sensing as well as posture control. Moreover, preflexes provided by the musculoskeletal system of the animal have been shown to stabilize locomotion (Jindrich and Full, 2002; Seipel et al., 2004; Proctor and Holmes, 2010).

Legged animals are able to compensate for small terrain irregulaties using their legs. However, significant irregularities, such as edges, can cause problems of instability due to the rigidity of the thorax. Thus, legged animals increase their mobility by having jointed body segments rather than a single rigid one. This biomechanical property is found in many insects, such as cockroaches and stick insects. (Ritzmann et al., 2004) observed that control of the two body joints provides significant support for obstacle negotiation in insects. Cockroaches use their body joint to support their locomotion over an object by bending the front body downwards (Ritzmann et al., 2004). This specific behavior can be referred to as body flexion. It allows the cockroaches to apply more vigorous propulsion forces. Even more importantly, it keeps the CoM near to the surface, so that the cockroach does not fall over backwards.

Generally, moving through complex landscapes may cause problems for legged animals due to irregularities, e.g., holes in the ground as well as steps. As mentioned earlier, legged animals are able to deal with such irregularities by alternating leg movements based on reflexes. The so-called searching and elevator reflexes observed in insects lead to effective rough terrain locomotion and climbing over obstacles (Cruse, 1979; Bässler, 1983; Pearson and Franklin, 1984). The searching reflex appears in situations in which a leg looses ground contact during stance phase, e.g., in a hole or a pit. The reflex then extends the respective leg to search for a foothold. This behavior has been found in several insects, including stick insects and locusts (Fischer et al., 2001). Moreover, insects are able to avoid hitting an obstacle with their legs triggered by the elevator reflex (Franklin, 1985). This reflex leads to a higher amplitude of succeeding leg swings.

The following section introduces a biologically-inspired, adaptive neural control mechanism for obstacle negotiation behavior in hexapod robots utilizing the aforementioned adaptive and reactive behaviors of insect climbing. As we will later show, this leads to effective and energy-efficient negotiation of a wide range of different obstacle heights.

## 3. Materials and methods

The six-legged walking machine AMOS II is employed as the experimental platform of this work. For practical reasons, we developed and tested our proposed control system first on a robot simulation framework called *LpzRobots* using an accurate, simulated model of AMOS II, and then we successfully transferred it to the real robot. Therefore, the next section describes its biomechanical setup and the simulation framework. Finally, we present the adaptive neural control mechanism for obstacle negotiation and its modules while some results are shown alongside to illustrate their operating principles.

### 3.1. Biologically inspired hexapod robot AMOS II

**Figure 1:**
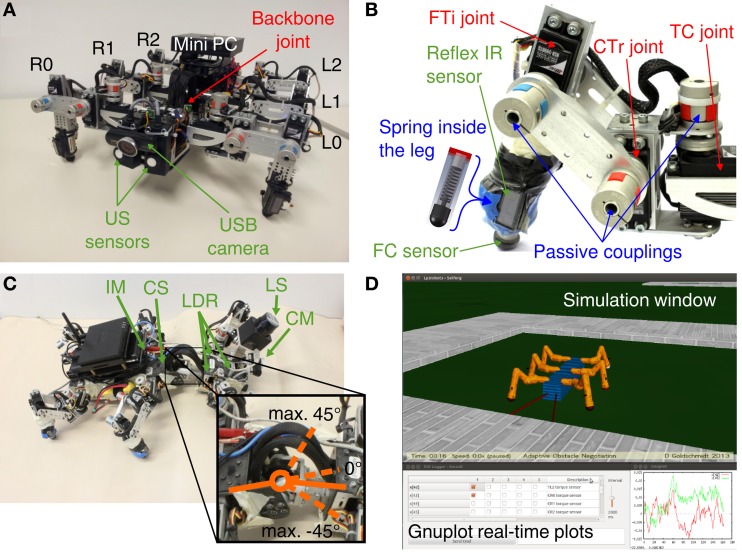
**Biologically-inspired hexapod robot AMOS II. (A)** Sensors and leg configuration of AMOS II (front view). **(B)** Motor, sensor and passive component configuration of an AMOS II leg (R0). **(C)** Example of flexion and maximum range of the backbone joint and additional sensors of AMOS II. **(D)** Robot simulation toolkit *LpzRobots* with GUI and real-time plots. Abbreviations: L0, R0 = left and right front legs, L1, R1 = left and right middle legs, L2, R2 = left and right hind legs.

The biomechanical and control design of the hexapod walking machine AMOS II (Figure 1) is biologically inspired by insects (Manoonpong et al., 2008a). One of the important characteristics of AMOS II is an actuated hinge joint called the backbone joint (BJ) connecting the two parts of its body enabling controlled flexibility in the thorax (Figures 1A,C). One of its three similar leg pairs is attached to the front body part and two are attached to the rear body part. The motion of the thoracal-coxal (TC) joint is limited in forward (+) and backward (−) directions, the coxal-trochanteral (CTr) joint elevates (+) and depresses (−) the leg and the femoral-tibial (FTi) controls extension (+) and flexion (−) of the third limb (Figure 1B). The 19 DC servomotors (three at each leg, one BJ) actuate the joints of AMOS II with a stall torque of 2.9 Nm at 5 V and are controlled in position mode. Additionally, the backbone joint torque is increased threefold by using a gear to accomplish a more vigorous motion. To perceive its environment, AMOS II has mounted 21 sensors in total: two ultrasonic (US) sensors attached to the front body part, six foot contact (FC) sensors for its legs, six reflex infrared (IR) sensors located at the front of its legs (Figure 1B), a built-in current sensor (BICS) and an inclinometer sensor (IM) inside the trunk, three light dependent resistors (LDR), a USB camera (CM), and a laser scanner (LS) at the front body part (Figure 1C). This large number of sensors enables the robot to generate various behaviors, such as obstacle avoidance and phototropism (Manoonpong and Wörgötter, 2009; Steingrube et al., 2010). Here only the US, FC, and IR sensors are employed for adaptive obstacle negotiation behavior. We use the robot simulation framework *LpzRobots* (Martius et al., 2010) based on the Open Dynamics Engine [ODE, see Smith (2006)] to simulate AMOS II (see Figure 1D) and to test the developed adaptive neural control before transferring it to the real robot. Note that the simulation uses the same physical parameters as the real robot and, therefore, control parameters obtained from the simulation can be directly tested on the real robot, leading to almost identical behavior. Additionally, it enables us to accurately predict the maximum obstacle or step height that the robot can overcome.

### 3.2. Control architecture for adaptive obstacle negotiation behavior

**Figure 2:**
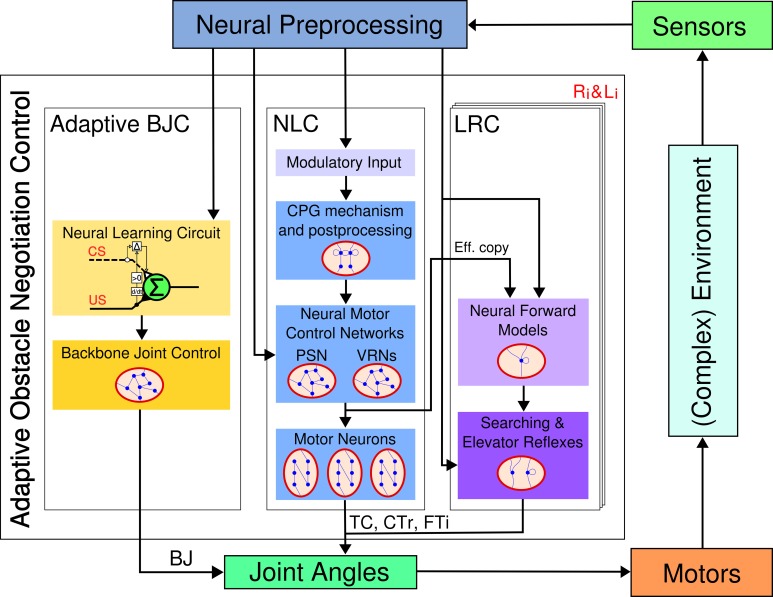
**Embodied closed-loop neural control architecture for adaptive obstacle negotiation behavior**. It is composed of an adaptive backbone joint control (BJC), a neural locomotion control (NLC), and six local leg reflex control (LRC) units (i = 0,1,2). While the BJC drives the backbone joint (BJ), the NLC and the LRC both control the TC, CTr, and FTi joints of each leg (see text for detailed description). Abbreviations are referred to Figure 1.

The proposed control architecture for adaptive obstacle negotiation behavior (see Figure 2) is composed of three major neural control modules: adaptive backbone joint control (BJC), central pattern generator (CPG)-based neural locomotion control (NLC), and local leg reflex control (LRC). The adaptive BJC combines a neural learning circuit, based on input correlation learning, and hierarchical, behavior-based neural control. The neural learning circuit enables AMOS II to perform adaptive behavior of the backbone joint. In principle, it learns to respond to a conditioned stimulus, here an ultrasonic signal related to a distance to an obstacle. The neural learning circuit output is fed into a recurrent neural network with five input neurons generating three different, hierarchical behaviors. Finally, the backbone joint is directly driven by a summation output neuron. The NLC generates frequency- and phase-modulated periodic signals controlling all leg joints resulting in various insect-like behavioral patterns for walking, including omnidirectional locomotion, turning, and a multitude of hexapedal gaits. The robot is therefore able to traverse different kinds of terrains in the maximum efficiency attained by the AMOS II system (Manoonpong et al., 2013b). The set of different behavioral patterns can be autonomously controlled by higher-order control structures based on input signals coming from exteroceptive sensors, such as a camera, a laser scanner, and ultrasonic sensors. The LRC allows AMOS II to effectively react to variations of terrain. To do so, it provides two efficient reflexes using proprioceptive (i.e., FC sensors) and visual feedback for rough terrain locomotion: the searching and the elevator reflex. The searching reflex is activated as soon as a FC sensor loses ground contact in the stance phase. The respective leg consequently starts to adapt its movements through extension to search for the ground. The elevator reflex prevents the leg from hitting an obstacle during swing phase by using an IR sensory input. Neural preprocessing is applied for noise elimination as well as functional signal modulation. All control networks apply a standard additive, non-spiking neuron model with a discrete-time updating frequency of approximately *f*_*TS*_ ≈ 27 Hz. The activity of a neuron *i* is given by

(1) $$a_{i}(t+1)=\sum_{j=1}^{n}w_{ij}\;\sigma_{j}\left(a_{j}(t)\right)+\Theta_{i},\;\forall i=1,\ldots ,n,$$

where *n* corresponds to the number of neurons, Θ_*i*_ is a constant bias term to the neuron *i*, and *w*_*ij*_ the synaptic weight of the connection from neuron *j* to neuron *i*. For the output of the neuron, we use different types of transfer functions here: the standard sigmoid σ_*i*_ (*a_i_*(*t*)) = (1 + exp(−*a_i_*(*t*)))^−1^, hyperbolic tangent σ_*i*_(*a_i_*(*t*)) = tanh(*a_i_*(*t*)), linear, and binary threshold transfer function. Input units are linearly mapped onto the interval [0, 1] (for sigmoid, linear, and binary threshold) and [−1, 1] (for hyperbolic tangent). Note that artificial neural networks are used here as robot control because they are conceptually close to biological systems compared to other solutions (Kalakrishnan et al., 2011). They can form as a modular structure where the entire controller consists of different functions as shown here. Additionally they also allow for the application of different off-line and on-line learning algorithms.

### 3.3. Neural preprocessing of sensory data

**Figure 3:**
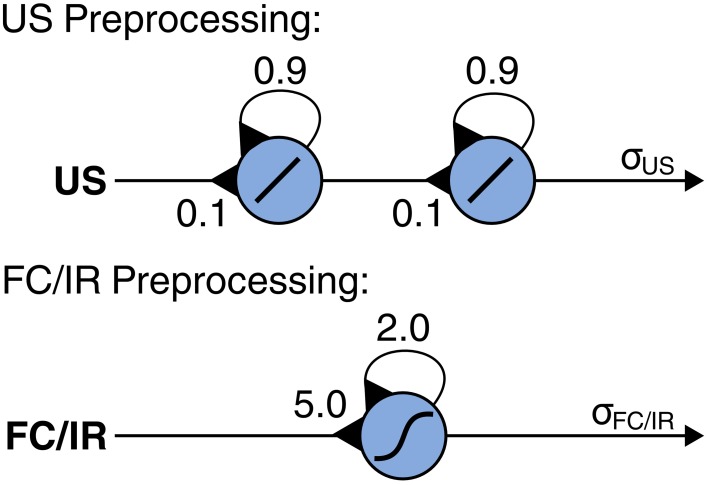
**Recurrent neural modules for signal preprocessing of ultrasonic (US), foot contact (FC), and infrared (IR) sensors**. US preprocessing applies two recurrent neurons using a linear transfer function where the incoming connection weights are set to 0.1 while the recurrent weight of each neuron is set to 0.9. FC and IR preprocessing modules both apply a single recurrent neuron using a hyperbolic transfer function where the incoming connection weights are set to 5.0 and the recurrent weights are set to 2.0. The neural parameters of all sensory preprocessing modules were chosen empirically.

Sensory data from the ultrasonic (US), foot contact (FC), and infrared (IR) sensors serve as input to generate adaptive obstacle negotiation behavior. For signal preprocessing of the sensory data, we apply neural modules with self-excitatory connections for non-linear filtering and hysteresis effects based on their dynamical properties [see Manoonpong et al. (2008b) for details about neural preprocessing of sensory signals]. Non-linear filtering suppresses unwanted noise (i.e., low-pass filtering) and shapes the sensory data, while hysteresis effects assure the neural activation to be present longer than the stimulus itself. This short-term memory capacity depends on the strength of the self-recurrent connections (Pasemann, 1993). For the US sensory preprocessing module, we apply a series of two single recurrent neurons where the weights were chosen empirically (Figure 3). As a result, the connection weights from the US input to the first recurrent neuron and from the first recurrent neuron to the second recurrent neuron are set equally to 0.1 while the recurrent weight of each neuron is set to 0.9. Using this setup, the module prolongs the activation of the sensory signal which is required to maintain control of the backbone joint for obstacle negotiation. For the FC and IR sensory preprocessing modules, we apply a single recurrent neuron for each module using the same neural parameters (Figure 3). The parameters were empirically adjusted. As a result, the connection weights from the FC input to its recurrent neuron and the IR input to its recurrent neuron are set equally to 5.0 while the recurrent weight of each neuron is set to 2.0. Using this setup, each module eliminates noise and shapes its sensory signal to a binary-like output [see also Steingrube et al. (2010); Manoonpong et al., (2013b) for details].

### 3.4. Adaptive backbone joint control

In this work, adaptive backbone joint control (BJC) has been developed for generating efficient obstacle negotiation behavior. It consists of two components: a neural learning circuit and a behavior-based, hierarchical neural network, also referred to as backbone joint control. While neural learning allows the robot to learn the association between a predictive (conditioned, CS) and a reflex stimulus (unconditioned, UCS), the BJC integrates sensory information from US and FC sensors to generate an output signal that directly drives the backbone joint.

#### 3.4.1. Neural learning circuit

**Figure 4:**
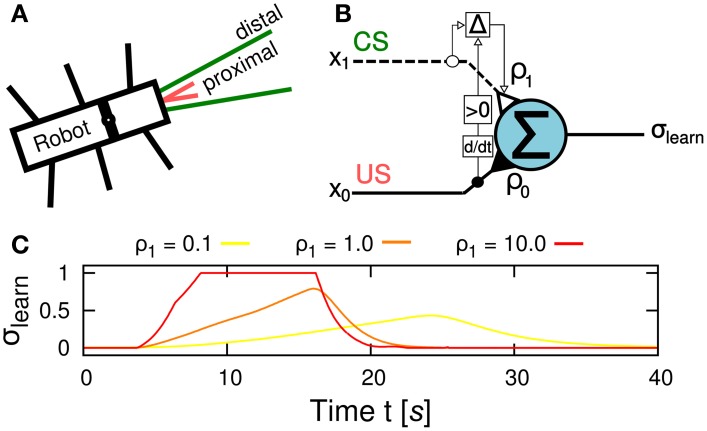
**Learning mechanism for insect-like, adaptive obstacle negotiation behavior. (A)** Sensory signal setup of the robot with proximal and distal signal ranges. **(B)** Neural circuit of the input correlation (ICO) learning rule for correlation-based learning. The correlation (depicted by Δ) of an early, reflex signal *x*_0_(*t*) a late, predictive signal *x*_1_(*t*) alters the weight of the plastic synapse ρ_1_(*t*) according to Equation 3 while the synaptic weight ρ_0_ is fixed. The output neuron applies a linear transfer function to generate the output signal σ_learn_. Note that the learning rule applies the positive time-derivative of *x*_0_ to change the plastic synapse ρ_1_(*t*). **(C)** Behavioral response to different values of ρ_1_. The ascent slope of the learner neuron output σ_learn_ controlling the BJ increases proportional to the strength of ρ_1_, thus, the higher the value the faster and stronger the leaning motion takes place. Note that the behavioral output also alters the perceived sensory input such that a faster response leads to an earlier cut-off, i.e., when the robot leans beyond the edge of the obstacle. As a result, the time interval in which the BJ is active gets shortened.

The learning goal in this study is to learn to efficiently negotiate an obstacle. This entails learning to tilt the front body part upwards early enough to avoid running into the obstacle. Preprocessed sensory signals coming from the US sensors at the front of the robot serve as input signals for the learning circuit. The US signal is divided into a proximal and a distal range (Figure 4A) leading to an early, predictive signal *x*_1_ and a late, reflex signal *x*_0_, respectively, as the robot approaches an object. While *x*_1_ is equal to the US signals fed into the circuit, *x*_0_ is only active when the US signals surpass a certain threshold Θ = 0.85 and is then mapped onto the interval [0,1]. The learning circuit employs a correlation-based, differential Hebbian learning rule called input correlation (ICO) learning (Porr and Wörgötter, 2006) to modify a plastic synapse ρ_1_ during learning (Figure 4B). It consists of an integrating output neuron that applies a linear transfer function. The neuron sums up the weighted input signals *x*_0_(*t*) (reflex signal) and *x*_1_(*t*) (predictive signal), thus the output signal σ_learn_ is given by:

(2) $$\sigma_{\text{learn}}(t)=\rho_{0}x_{0}(t)+\rho_{1}(t)x_{1}(t).$$

The synaptic weight ρ_0_ = 0.1 is set to be constant. The output signal σ_learn_ is fed into the BJC to control the leaning behavior of the robot which is described below. Thus, the strength of ρ_1_ is proportional to the BJ motor output signal (Figure 4C). The ICO learning rule alters the plastic synapse ρ_1_(*t*) as follows:

(3) $$\frac{d\rho_{1}(t)}{dt}=\mu x_{1}(t)\frac{dx_{0}(t)}{dt}.$$

Note that only positive changes of *x*_0_ are applied to modify the weight. At the beginning, ρ_1_ is set to zero and therefore, the leaning behavior is weakly controlled by the reflex signal. As a result, the robot leans too late to avoid hitting the obstacle. Consequently, the robot starts to turn away from the obstacle. Due to the correlation of *x*_0_ and *x*_1_, the plastic synapse ρ_1_ increases with every climbing trial resulting in a stronger response of leaning behavior to the predictive signal. Consequently, the robot leans earlier to avoid the occurance of the reflex signal. After a finite number of climbing trials, the plastic synapse ρ_1_ converges toward a value for which the leaning behavior avoids the reflex signal completely.

#### 3.4.2. Backbone joint control

**Figure 5:**
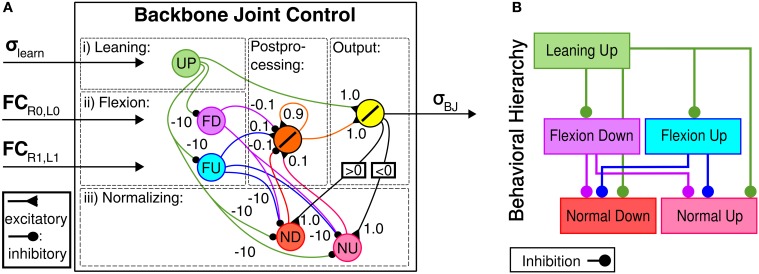
**Backbone Joint Control (BJC). (A)** Neural module for BJC. It receives preprocessed sensory input signals from ultrasonic (US) and foot contact (FC) sensor. Five input neurons are connected to an output neuron which signal σ_*BJ*_ directly drives the backbone joint. **(B)** Behavior-based, hierarchical topology of BJC. Lower ordered behaviors are surpressed through inhibitory synapses.

The behavior-based, hierarchical BJC is applied to enhance obstacle negotiation of the robot by emulating thoracic joint behaviors observed in cockroaches. It is a recurrent neural network that consists of five input neurons, a hidden, postprocessing neuron and a summation output neuron that directly drives the backbone joint and has a behavior-based, hierarchical topology (see Figures 5A,B). The behavior-based approach of the BJC is inspired by key behaviors for efficient obstacle negotiation as observed in insects. Three different behaviors are induced by the BJC: Leaning, flexion and normalizing. Leaning behavior emulates a behavior observed in stick insects in which the animal leans its front body part upwards before approaching an obstacle (Schütz and Dürr, 2011). This leads to a positive body-substrate angle which is required for an effective placement of the front tarsi onto the obstacle. Note that leaning behavior is used to approach the obstacle instead of rearing behavior of cockroaches resulting in significant instabilities of walking for our robot. The leaning neuron (UP) receives the output σ_learn_ from the neural learning circuit described above and generates an output signal using a linear transfer function. Flexion behavior emulates body flexion of insects (Ritzmann et al., 2004) in order to increase the efficiency of traversing surface irregularities, including edges and depressions. It is generated by two neurons SD and SU for downward and upward movements, respectively. They receive input FC sensory signals from the front (for SD) and the middle leg pair (for SU), respectively. Both neurons serve as an AND gate using a binary threshold transfer function. By this means, these neurons are only activated when both legs of the respective leg pair are in the air. Consequently, flexion behavior allows the robot to contain stability and effective locomotion on irregular terrain. Normalizing behavior drives the backbone joint to normal position while walking on even terrain, such as after obstacle negotiation. This behavior is controlled by two neurons ND and NU for downward and upward movements, respectively. They receive recurrent feedback from the output neuron of the neural network and apply a threshold transfer function. The binary output signals of the neurons for flexion and normalizing behavior are fed into a postprocessing neuron applying a linear transfer function that serves as a lowpass filter to smoothen the output signal. Due to the hierarchical structure of the controller the occurence of higher-ordered behaviors suppresses those of lower-ordered behaviors. In neural networks, this is done by neural inhibition through negative synaptic weights. The described topological properties of the behavior-based, hierarchical BJC result in both the maximum energy efficiency achieved by the AMOS II system (see Figure 14 shown in the Experimental results section) and versatile body joint behavior for obstacle negotiation.

### 3.5. Neural locomotion and local leg reflexes

**Figure 6:**
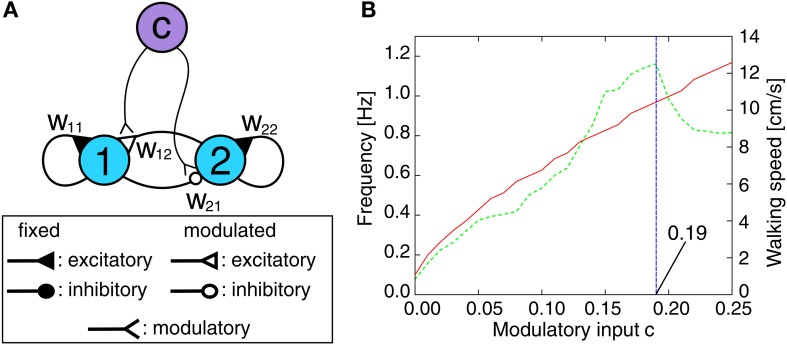
**Neuromodulated CPG mechanism. (A)** CPG circuit diagram for periodic pattern generation. The synaptic weights *w*_12_ and *w*_21_ are modulated by an extrinsic control input *c*, i.e., *w*_12_ = 0.18 + *c* = −*w*_21_. The fixed synaptic weight are set to *w*_11_ = *w*_22_ = 1.4. **(B)** The eigen frequency of the resulting CPG output signals (red solid line, left y-axis) and the corresponding walking speed of AMOS II (green dashed line, right y-axis) with respect to the modulatory control input *c*. The sample interval of *c* is Δ*c* = 0.01. For negative values of *c* the network dynamics exhibits solely fixed point attractors instead of periodic or quasi-periodic ones. Note that the updating frequency is approximately *f*_*TS*_ ≈ 27 Hz which corresponds to a time step interval of *t* ≈ 0.037 s.

Controlling only the backbone joint is not sufficient for successful obstacle negotiation. This section briefly describes the neural locomotion control (NLC) and the local leg reflex control (LRC) as additional modules required for adaptive obstacle negotiation behavior. As presented in (Manoonpong et al., (2008b), it is composed of a neuromodulated CPG mechanism including postprocessing, neural motor control networks and motor neurons with a delay line mechanism that are finally sent to all leg joints of AMOS II. By generating a large variety of periodic output signals without relying explicitly on sensory input, the CPG mechanism provides a broad repertoire of insect-like walking gaits. It is a fully connected, two-neuron circuit (Figure 6A). The activity function *a*_*i*_ for both neurons *i* ∈ {1, 2} is defined as shown in Equation 1 and the tanh function is applied as the transfer function generating the output signal σ_*i*_. The connections are defined by two fixed synapses with *w*_11_ = *w*_22_ = 1.4 as default weight generating periodic or quasi-periodic attractors (Pasemann et al., 2003) and two synapses *w*_12_ = −*w*_21_ = 0.18 + c modulated by an extrinsic input given by the control parameter *c* (see Figure 6A). By increasing *c*, the output signal frequency and thus, the walking speed increases proportionally up to a certain limit (*c* ≤ 0.19, see Figure 6B). This restriction is given by the limited response frequency of the motors of AMOS II. Due to neural motor control and the delay line mechanism of the motor neurons (described below), AMOS II is able to perform different walking gaits depending on the value of *c* (e.g., 20 patterns). In order to show the adaptability and performance of our control system, we will compare six different gaits: wave (*c* = 0.03), tetrapod (*c* = 0.06), caterpillar (*c* = 0.09), intermixed (*c* = 0.12), slow (*c* = 0.15) and fast tripod (*c* = 0.18) gaits. The selected slow wave gait is more stable for climbing compared to another faster wave gait generated by *c* = 0.04. According to this using the slow wave gait leads to the highest climbable obstacle (i.e., 15 cm, see the Experimental results section) which cannot be achieved by the faster wave gait. Thus, we use here the slow wave gait as one of selected gaits for experiments while the other gaits are arbitrarily chosen without being tuned for a particular surface. The CPG outputs are transmitted to motor neurons through its postprocessing and neural motor control networks. The postprocessing unit is for slightly shaping the CPG signals for smooth movements. The motor control networks perform further phase and frequency modulation of the postprocessed CPG signals enabling AMOS II to walk omnidirectionally, e.g., walking backwards and sidewards as well as turning in each direction. They consist of three generic feed-forward networks: a phase switching network (PSN) and two velocity regulating networks (VRNs). They have been developed using standard neural learning algorithms (i.e., backpropagation learning). The PSN switches the phase of the periodic signals controlling the motors at the CTr and FTi joints. This allows us to control lateral movements of the robot [see Manoonpong et al. (2008a) for details]. The VRNs control the TC joints on each side. The impact of the VRNs on the locomotion can be modulated by exteroceptive sensors to induce obstacle avoidance behavior. In general, these inputs affect the velocity of the right- and left-sided TC joints by scaling the amplitude of all signals. This allows for transversal locomotion as well as turning [also see Manoonpong et al. (2008a) for details]. Finally, the output signals from the motor control networks are transferred to motor neurons through delay lines. These delay the output signal for each joint type (i.e., TC, CTr, and FTi) about τ = 0.8 s for each following ipsilateral leg (from hind to front) while there is an additional delay τ_*L*_ = 2.4 s between the left and right side. Note that these delays are fixed and independent of the CPG signals.

**Figure 7:**
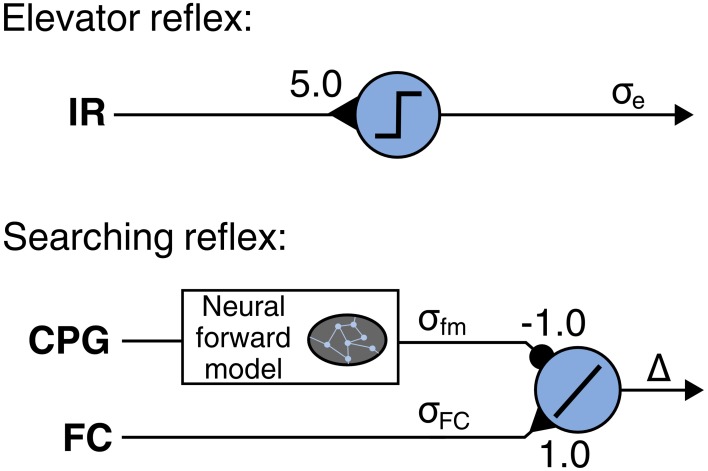
**Neural modules for local leg reflexes: searching and elevator reflex**. A neural forward model is applied to determine expected foot contact signal from leg motor pattern provided by the CPG. The additive neuron accumulates positive errors which cause vertical shifting of the CTr and FTi signals, resulting in searching reflex. A preprocessed infrared reflex signal are fed into a neuron using a linear threshold transfer function. The threshold is set to 0.01. The output signal σ_*e*_ shifts the CTr and FTi signals upwards. As a result, the leg is elevated. Note that each leg has its own searching and elevator reflex modules.

Negotiating obstacles requires adaptations of leg movements due to irregularities of the terrain. In order to tackle this issue, we employ the LRC that emulates two reflexes observed in insects. The first reflex is the searching reflex in which the leg searches for a foothold when the foot has no ground contact, thus the foot contact signal σ_fc_ does not match the leg motor pattern. This reflex is implemented by using a neural module (Figure 7) including a neural forward model (Manoonpong et al., 2013b). The model transforms a motor command from the CPG (efference copy) into an expected foot contact signal σ_forward_ for normal walking [see Manoonpong et al. (2013b) for more details]. The output signal of the module is given by Δ = σ_fc_ − σ_forward_ and can be referred to as an error. The actual reflex is enforced by vertical shifting of the CTr and FTi signals using the accumulation of significant, positive errors: Δ_+_ = ∑_*t*_|Δ(*t*)|; ∀ Δ(*t*) ≥ 0.15. Consequently, the CTr/FTi signals are shifted when an error occurs, such as when the respective leg searches for a foothold. The second reflex causes a leg elevation after the leg touches an obstacle during the swing phase. This reflex is called elevator reflex. It is induced by the infrared (IR) reflex sensors at the front of each leg (Figure 7). Using this sensory data as an input signal, a threshold neuron provides an output signal σ_*e*_ which shifts the CTr/FTi signals upwards, i.e., the leg is elevated. Note that this elevator reflex only occurs in the swing phase.

## 4. Experimental results

In order to evaluate the performance and robustness of the proposed learning mechanism and control system, we carried out six experiments using the simulated robot for the first four experiments and the real robot for the final two experiments.

**Figure 8:**
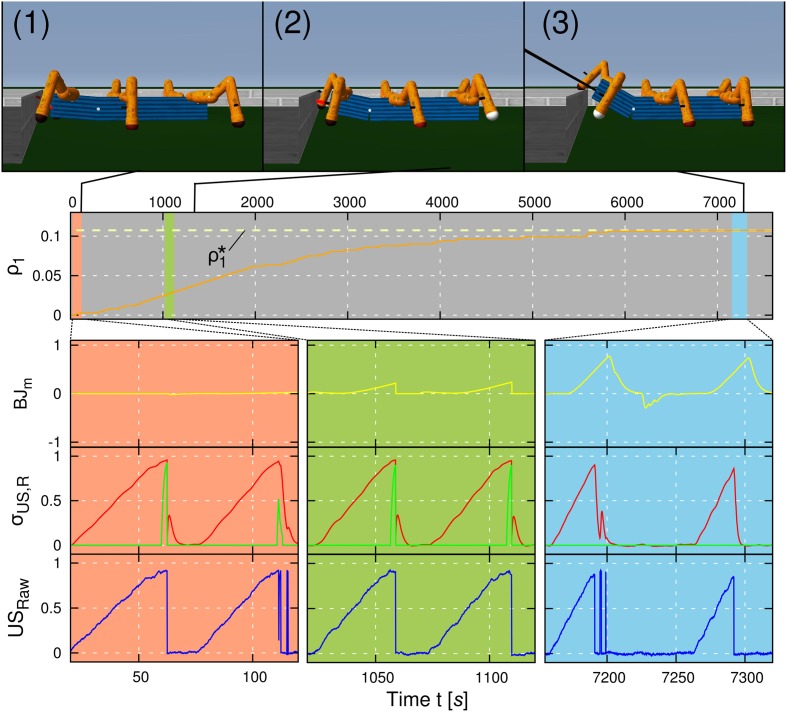
**Learning curve of a negotiation of an obstacle with a height of 8 cm**. The weight ρ_1_ (orange line) changes according to the ICO learning rule, i.e., due to correlations of the predictive signal (red line) and the reflex signal (green line). In the beginning of the experiment, the backbone joint (*BJ*_*m*_, yellow line) does not exhibit any behavior because of a small weight ρ_1_ (see salmon colored area, photo 1). With an increasing weight ρ_1_, the amplitude as well as the slope of the backbone joint motor output increases (see olive colored area, photo 2). After learning, ρ_1_ has converged toward a value ρ^*^_1_ (dashed line) for which the robot generates an optimal backbone joint motion, i.e., an optimal leaning behavior (see sky blue colored area, photo 3). The convergence is due to the fact that the reflex signal is avoided such that it does not occur after learning. Raw data (*US*_raw_, blue line) coming from the ultrasonic (US) sensors is also shown. Note that all graphs illustrated in this figure correspond to the right US sensor. The learning curve of signals coming from the left US sensor shows a similar behavior. The learning rate is set to μ = 0.01.

The first experiment is carried out to illustrate the convergence of the applied learning mechanism for negotiating an obstacle using a wave gait (*c* = 0.03). The experimental result is shown in Figure 8. We placed AMOS II in a square surrounded by an 8 cm high obstacle with a fixed initial distance *d* = 0.75 m. The task of AMOS II was to approach and negotiate the obstacle. The learning goal of AMOS II during this task was to optimize its backbone joint motion with respect to the synaptic plasticity of the learner neuron. For the rest of this article this experimental setup is referred to as the convergence trial. As described in the previous section, the plastic synapse ρ_1_ of the learner neuron increases due to the correlation of the predictive and the reflex signal provided by the US sensors. Initially, ρ_1_(*t* = 0) is set to zero resulting in a diminutive leaning behavior of the BJ driven only by the reflex signal (Figure 8, salmon colored area). Thus, for this height, the robot is unable to negotiate the obstacle and hits it; thereby activating the reflex signals. As a consequence, for practical reasons in the first and second experiments, the robot is automatically reset to its initial position and then reapproaches the obstacle. However, instead of resetting the robot position, the reflex signals can be used to drive the robot to avoid the obstacle; enabling it to learn to overcome obstacles in an autonomous way without interference (see Video S1). During learning, the plastic weight ρ_1_ increases and therefore, the robot leans the BJ faster and with a larger amplitude (Figure 8, olive colored area). At some point, the BJ motion is sufficient to negotiate the obstacle successfully (Figure 8, sky blue colored area). Because the occurence of the reflex signal *x*_0_(*t*) vanishes over time, the weights converge at finite time instant *T*, i.e., ρ_1_ (*t* = *T*) ≡ ρ^*^_1_ = const. Our convergence time criterion is set to *t*_*c*_ = 900 s without any change of ρ_1_. Here the weights converge after approx. 7500 s. Note that the time required to let the weights converge is related to the learning rate μ which is here set to 0.01. We have chosen a small learning rate to avoid overshooting a possible optimum for ρ_1_.

**Figure 9:**
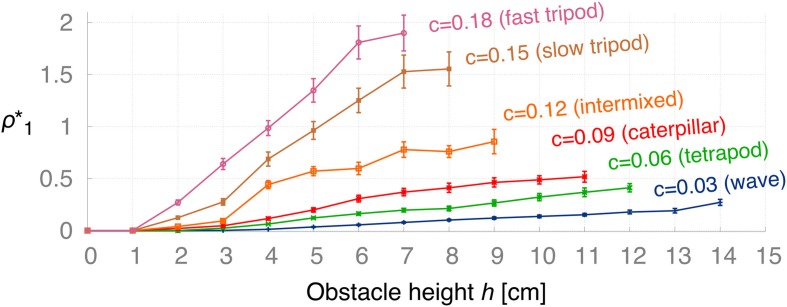
Average learned weights ρ^*^_1_ (±SD) with respect to the obstacle height *h* for different gaits: wave (*c* = 0.03), tetrapod (*c* = 0.06), caterpillar (*c* = 0.09), intermixed (*c* = 0.12), slow tripod (*c* = 0.15), and fast tripod (*c* = 0.18) gait. For increasing *h* and *c* the learned weights converge to higher values, respectively. For the more unstable, faster gaits the learning algorithm does not converge due to instabilities during climbing. However, these non-convergences correspond to the maximum obstacle height that the robot is able to achieve for given gaits (see Figure 12).

**Figure 10:**
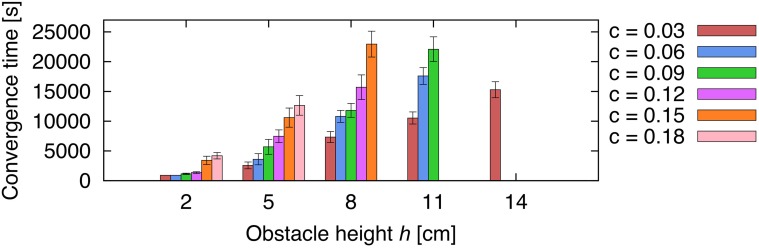
**Average convergence times for five selected heights using different gaits**. Five different obstacle heights (*h* = 2, 5, 8, 11, 14 cm) were selected using the set of gait parameter as described in Figure 9 to compare the average convergence times (±SD) of 20 learning trials.

In order to deal with changes in certain conditions, such as the obstacle height and the walking speed of the robot, the ICO learning rule adapts the backbone joint behavior accordingly by converging to different weights. The second experiment aims to determine learned weights ρ^*^_1_(*h*, *c*) after convergence of the learning algorithm with respect to obstacle height *h* and modulatory input *c* defining the walking gait. For each the 15×6 parameter pairs of *h* = 1, 2, 3,…, 15 cm and *c* = 0.03, 0.06, 0.09, 0.12, 0.15, 0.18, we ran 20 convergence trials to obtain the learned weights (ρ^*^_1_)_*k*_. Figure 9 presents the averaged learned weights $\rho_{1}^{*}=\frac{1}{20}\sum_{k=1}^{20}{\left(\rho_{1}^{*}\right)}_{k}$ obtained from this experiment. The results show that for higher obstacles *h*, the learning algorithm converges at larger values ρ^*^_1_ meaning that the robot must apply a stronger backbone joint motion to successfully negotiate the obstacle. The same applies for higher gait parameters *c* (i.e., faster walking speed). In principle, ICO learning is able to converge for any of the given parameters because it avoids the unwanted reflex signal by responding earlier due to the predictive signal. However, Figure 9 shows that ICO learning does not converge in a finite amount of time for some of the parameter pairs, i.e., higher obstacles and faster gaits. This is due to dynamical perturbations caused by the tilting of the front body during certain gaits. These instabilities lead to random occurrences of the reflex signal during a trial, and thus, to weight changes of the plastic synapse. This effect is rather small for the more stable gaits such as the wave gait but occurs more often for faster gaits. Furthermore, the non-convergence of ICO learning seems to coincide with the maximum climbing capabilities for given parameters (h,c) as shown later in Figure 12. Figure 10 shows the average convergence times of 20 learning trials for selected obstacle heights (*h* = 2, 5, 8, 11, 14 cm) and gait parameters as described above. At 2 cm, the weights for the slower gaits (*c* = 0.03, 0.06, 0.09, 0.12) converge approximately at the criterion time (*t*_*c*_ = 900 s) while for the faster gaits (*c* = 0.15, 0.18) the weights take a longer time to finally stabilize. This is due to the instabilities mentioned above causing the reflex signal to occur randomly. For higher obstacles, convergence times increase due to larger weights which are required to tilt the backbone joint with a larger amplitude to surmount the obstacles. The convergence times are depending on the learning rate chosen. The learning rate used for this experiment is rather small (μ = 0.01) to avoid overshooting of optimal weights. Also, note that the convergence criterion has been chosen very conservatively to ensure learning the optimal weights.

**Figure 11:**
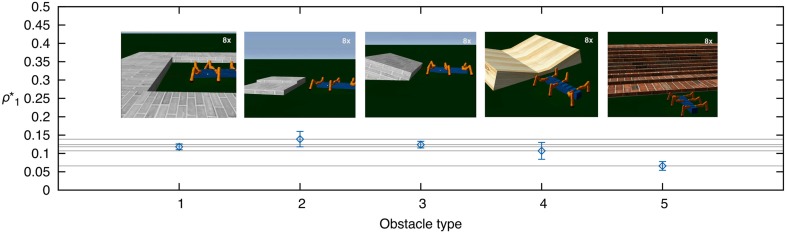
**Average learned weights for complex obstacles**. We tested five different obstacle types (see Video S2) and measured the average learned weights ρ^*^_1_ (±SD) for 5 trials each.

The third experiment exemplifies the generic use of our adaptive obstacle negotiation control for more complex obstacles. We tested obstacle negotiation learning of five different obstacle types (see Video S2). To demonstrate weight convergence, we set the learning rate to μ = 0.1. Figure 11 shows the resulting average learned weights ρ^*^_1_ (±SD) for 5 trials for each obstacle. The results show that the robot is able to deal with more complex obstacles. The learned weights approximately correspond to values of the respective heights required to overcome the obstacle (see the Discussion section for details).

**Figure 12:**
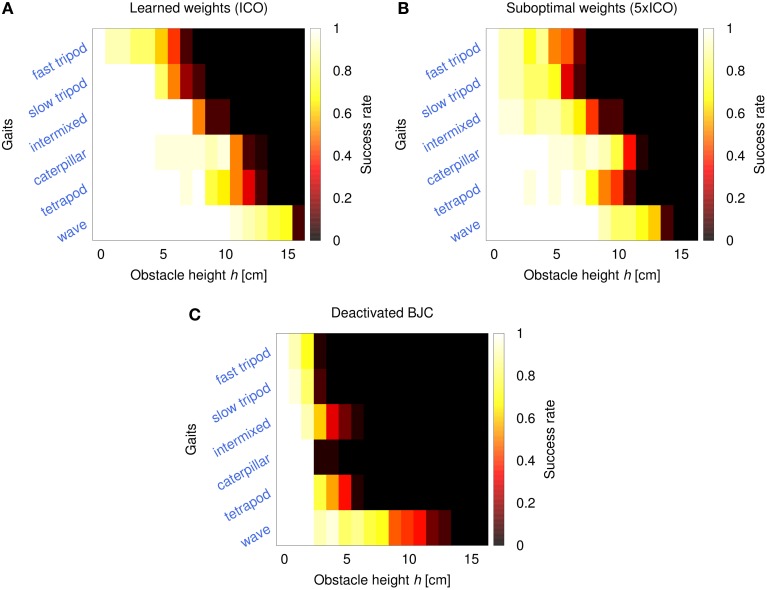
**Performance heatmaps of AMOS II obstacle negotiation with respect to obstacle height *h* and gait parameter *c* using three different control configurations. (A)** Activated BJC with learned weights (ICO), **(B)** activated BJC with suboptimal weights (5× ICO), **(C)** and deactivated BJC. The performance is measured by the success rate of 20 trials. The robot is successful by traversing a 1 m distance in a certain time (*t* < 180 s) with an obstacle on the way.

The fourth experiment investigates AMOS II performance in obstacle negotiation comparing three different configurations: activated BJC using weights from ICO learning, activated BJC using suboptimal weights (i.e., five times larger than ICO), and deactivated BJC. The performance is measured by the success rate after 20 trials. In each trial, the robot has to traverse a distance of 1 m with an obstacle placed in the middle. Here, success means that the robot surmounts the obstacle without triggering the reflex signal and reaches the 1 m goal position in a certain period of time (*t* < 180 s). The results are shown in Figure 12. Using the weights from ICO learning, the robot is able to climb obstacles with a maximum height of 15 cm in an effective way (i.e., success rate >0.5). This maximum height is achieved using a wave gait. For faster gaits, the robot shows decreasing climbing performance but it is still able to surmount high obstacles (5−10 cm). The manually increased weights of the second configuration lead to suboptimal backbone joint behavior which decreases the performance in obstacle negotiation. The maximum obstacle height the robot is able to achieve using these suboptimal weights decreases by up to 2 cm for some of the gaits (e.g., 13 cm for the wave gait). Finally, deactivating the BJC leads to rapidly decreasing performance in obstacle negotiation. Although the robot is still able to surmount lower obstacles through local leg reflexes, the effect of the disabled BJC prevents the robot from overcoming higher obstacles.

**Figure 13:**
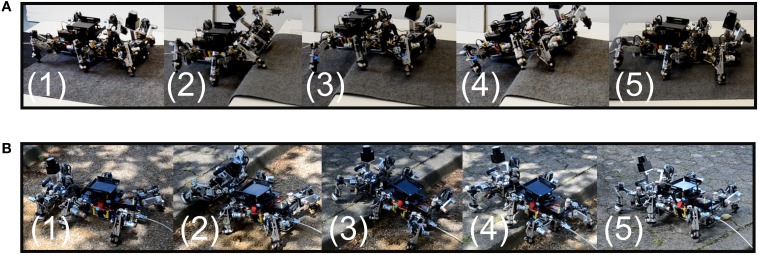
**Obstacle negotiation behavior in hexapod robot AMOS II over large obstacles in an (A) indoor and (B) outdoor environment**.

In the fifth experiment, we aim to show that the control system with learned parameters can be successfully transferred to the real robot AMOS II to generate obstacle negotiation behavior. To do so, we connected the robot to a PC through a serial interface. We placed the robot in front of an adjustable obstacle. Applying the learned weights ρ^*^_1_ (*h*, *c*), the robot is able to overcome various obstacles in a wave gait, including an 11 cm high obstacle as shown in Figure 13A. AMOS II approached the obstacle (photo 1) and tilted its backbone joint upwards as the US sensors detected the obstacle (photo 2). The backbone joint moves back into normal position when AMOS II's front leg is placed on top of the obstacle (photo 3). Note that this behavior is due to the lowpass filtered US signals driving the leaning behavior of the BJC. Downward flexion of the backbone joint and local leg reflexes support the stability in climbing behavior (photo 4). Finally, the robot surmounts the obstacle successfully (photo 5). The highest climbable obstacle that the robot can achieve has shown to be 13 cm using a wave gait (see Video S3). Moreover, we tested the robot in an outdoor environment. Figure 13B shows the robot climbing up a curb having a height of 11 cm. Besides climbing up an obstacle, the robot is also able to climb down from an obstacle as shown in Video S3.

**Figure 14:**
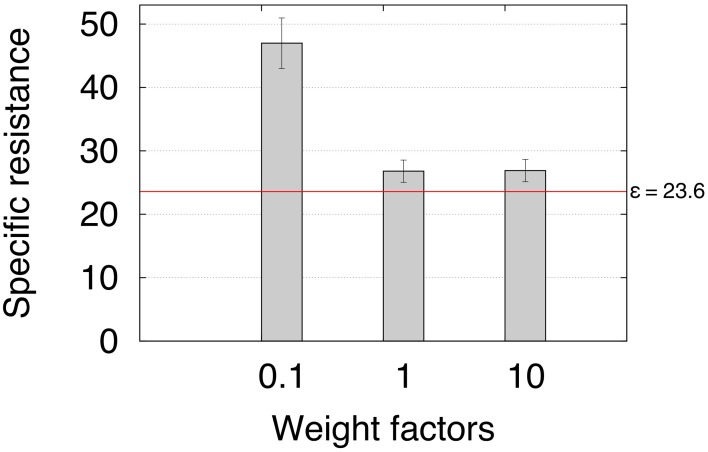
**Average specific resistance for the three different weight factors**. The specific resistance has been determined by measuring power consumption and travel time (see Table 1) of a 1 m distance with an obstacle on the way. The results suggest that a certain degree of backbone joint motion is necessary to produce energy-efficient obstacle negotiation behavior. The red line denotes the specific resistance for walking a 1 m distance without an obstacle in the way.

Finally, the sixth experiment investigated the energy efficiency of adaptive backbone joint control for obstacle negotiation in terms of specific resistance (Gabrielli and Von Kármán, 1950). We multiply the ICO learned weights ρ_1_ by three different weight factors ω = 0.1, 1 and 10 to investigate the specific resistance during climbing. The factors are used to compare the results for the weights obtained from ICO learning (ω = 1) with suboptimal weights (ω = 0.1, 10) The specific resistance is given by:

(4) $$\varepsilon =\frac{UI}{mgv},$$

where *I* is the average electric current in amperes measured by the Zap 25 BICS, *U* = 5 V is the supply voltage to the motors, *mg* = 56.9 N is the weight of the robot, and *v* is the walking speed of the robot (m/s). The walking speed is determined by the time *t* the robot needs to travel a distance of 1 m with an obstacle in the way. The obstacle used has a height of 11 cm. The carpet covering the obstacle forms a slope to ensure that the robot is still able to negotiate the obstacle, despite having weights smaller than those from ICO learning. This way we are able to compare the energy efficiency for the three configurations. Note that AMOS II can still overcome obstacles regardless of its material or slope as shown in Figure 13 and Video S3. For each weight, we repeated the experiment three times. Table 1 shows the average electric energy consumption *Pt* (±SD) in Ws. In addition, we provided the energy consumption of walking a 1 m distance without an obstacle in the way. The resulting average specific resistances ε (±SD) with respect to the weight factors (Figure 14) show that a certain degree of backbone joint behavior (i.e., ω ≥ 1) requires less energy and produces a much faster locomotion, allowing energy-efficient obstacle negotiation. The red line indicates the specific resistance ε = 23.6 for walking a 1 m distance without an obstacle in the way. Despite no significant differences between the specific resistances of ω = 1 and ω = 10, we have shown that the maximum climbable obstacle height decreases for weights considerably larger than the ones obtained from ICO learning (Figure 12).

**Table 1:** **Average electric energy consumption for climbing trials using three different weight factors and walking without an obstacle in the way**.

**ω**	**0.1**	**1**	**10**	**no obstacle**
*Pt* [Ws]	2673.15 ± 226.55	1524.44 ± 100.04	1529.96 ± 100.45	1341.50 ± 88.73

## 5. Discussion

In this work we introduced neural learning and control mechanisms for adaptive obstacle negotiation behavior in hexapod robots. The learning mechanism employs a differential Hebbian learning rule, called ICO learning. It strengthens a plastic synapse based on the correlation of temporal input sequences. The strength of the plastic synapse corresponds to the behavioral response of the active backbone joint of a hexapod robot. Here the hexapod robot AMOS II was used as our test bed. However, the learning and control mechanisms can be transferred to other hexapod robots (described below). Once the synapse has been established, it transmits a proximal, predictive signal to drive the backbone joint for negotiating an obstacle. This way, the robot achieves adaptive climbing behavior through associative learning and can deal with unknown or changing external environmental and internal conditions. Here, these conditions are given by different obstacle heights (environmental changes) and different robot walking speeds of the robot (internal changes) determined by a gait parameter of neural control. The weights of the plastic synapse converge in finite time for a large variety of these conditions leading to stable behavior. As a result, the behavioral response corresponding to the learned weights leads to a successful and efficient negotiation of given obstacles. Hence, the proposed neural controller generates robust obstacle negotiation behavior enabling the robot to autonomously overcome a wide range of different obstacles with different gaits. Furthermore, it is flexible enough to deal with more complex obstacle shapes as shown in Video S2.

Several correlation-based learning rules have been proposed for generating complex, adaptive locomotor behavior in a mobile robot (Grossberg and Schmajuk, 1989; Verschure et al., 1992; Verschure and Voegtlin, 1998; Porr and Wörgötter, 2003, 2006; Manoonpong and Wörgötter, 2009; Kulvicius et al., 2010; Kirwan et al., 2013). For example, the Distributed Adaptive Control (DAC) proposed by Verschure (Verschure et al., 1992) learns the association of an UCR to a conditioned stimulus and employs distal and proximal sensors installed on a robot for generating adaptive behavior, such as obstacle avoidance. The DAC learning rule is based on homosynaptic plasticity. This way, it requires an inhibition of conditioned stimuli to compensate for autocorrelation signals. Without the inhibition, weights can diverge. In contrast, ICO learning used here is based on heterosynaptic plasticity where this learning mechanism leads to weight stabilization as soon as a reflex signal (e.g., proximal sensory signal) has been successfully avoided. As a result, we obtain behavioral and synaptic stability at the same time without any additional weight-control mechanisms, like the inhibition mechanism used in the DAC. In addition, ICO learning allows implementation of fast learning [i.e., one shot learning (Porr and Wörgötter, 2006)]. While it has been previously applied for goal-directed behaviors (Porr and Wörgötter, 2006), obstacle avoidance behavior (Kulvicius et al., 2010; Kirwan et al., 2013) and escape behavior (Manoonpong and Wörgötter, 2009), it is for the first time here applied to obstacle negotiation. The basic physical components required for obstacle negotiation learning using our control scheme on a walking robot are a proximity sensor, such as an infrared sensor, an ultrasonic sensor, a laser range finder, or antennae, and an actuated body joint for tilting the front body part upwards and downwards. The adaptive control of a body joint basically increases the performance in obstacle negotiation and reduces the mechanical load acting on the legs whilst climbing. Although our results are presented on AMOS II, the controller can be also transferred to other walking robots, such as SpaceClimber (Bartsch et al., 2012) and Whegs™ II (Lewinger et al., 2005) which have the required basic physical components (i.e., actuated body joint and proximity sensor). However, other robots with actuated body joints, such as HECTOR (Schneider et al., 2012) and MechaRoach II (Wei et al., 2005), will require an implementation of a proximity sensor to apply our adaptive backbone joint control.

The coordination of obstacle negotiation behavior generated by our neural controller is based on neuroethological observations of insects such as cockroaches and stick insects. Although both individuals share certain key behaviors in solving this task, there are also significant differences in locomotion due to different morphologies. Stick insects generally walk and climb with a high center of mass, i.e., high ground clearance (Bässler, 1967; Graham, 1972; Cruse, 1976) and they perform upward tilting of their prothorax body segment to initiate a targeted reach-to-grasp movement of the front leg during climbing (Schütz and Dürr, 2011); while cockroaches exhibit low ground clearance for agile and energy-efficient locomotion and they extend their front and middle legs for rearing up the body and raising their center of mass by tipping on top of the obstacle (Watson et al., 2002). Hereby we combine these effective key behaviors for a hexapod robot, such as AMOS II. Specifically, we design the locomotion control for walking with low ground clearance followed the cockroach walking strategy. This leads to more stable and energy-efficient locomotion (Manoonpong et al., 2013b). For climbing behavior combining stick insect and cockroach strategies, the BJC drives the body joint to tilt a front body upwards and performs the reach-to-grasp movement in concert with the searching leg reflex as stick insects do. In order to lift the center of mass on top of the obstacle the searching leg reflex also controls the CTr and FTi joints, respectively, to extend the middle legs after the first foot contact is made on top of the obstacle. This behavior is related to the rearing and rising behaviors observed in cockroaches. Moreover, when traversing an edge, the BJC flexes the body joint downwards in resemblence with the behavior of stick insects and cockroaches (Ritzmann et al., 2004; Schütz and Dürr, 2011). Taken together this work suggests a biologically-inspired approach emulating insect climbing and locomotion behavior to solve a complex task, such as negotiating obstacles, in artificial legged systems. As mentioned earlier, insects use their antennae to detect and to evaluate obstacles (Harley et al., 2009). The hereby extracted tactile information serves as cues for controlling adaptive motor behaviors during climbing. These are mainly determined by the distance from the obstacle being the main parameter for the initiation of negotiation behavior. In an artificial walking system, such as AMOS II, ultrasonic sensors installed at its front can be used to perceive the distance to an obstacle and responds to these stimuli by generating adaptive backbone joint behavior for effective obstacle negotiation. Nevertheless, the underlying learning mechanisms applied for adaptive motor behavior for obstacle negotiation in insects still leaves an open question.

We compared the success rate of obstacle negotiation using the BJC with learned weights, with suboptimal, larger weights and deactivated BJC to show the effectiveness of the adaptive BJC for this task. It has been shown that the adaptive BJC is most effective for very high obstacles where our test bed AMOS II achieves robust negotiation of obstacles with a maximum height of 15 cm in simulation and 13 cm in a real environment. This height is approximately 85 and 75%, respectively, of the robot's leg length given by the length of the femoral and tibial leg segments. This measure ensures an appropriate comparison with other state-of-the-art, legged robots performing obstacle negotiation. The hexapod robots Gregor III (Arena and Patané, 2011) and SpaceClimber (Bartsch et al., 2012) achieved approximately 70%, and the quadrupedal LittleDog (Kalakrishnan et al., 2010) accomplished about 60% of its leg length. Other hybrid wheel-legged robots (i.e., one DOF per leg) such as Whegs™ II (Lewinger et al., 2006) and Rhex (Saranli et al., 2001) were able to overcome higher obstacles relative to their respective leg lengths. However, their climbing behavior is not comparable to climbing behavior of insects like stick insects and cockroaches. In addition, reducing the number of DOFs also decreases the mobility of the robot for, e.g., omnidirectional locomotion including sideways walking (Manoonpong et al., 2008a). Most of the aforementioned robots utilize parts of the strategies described in this work. Many studies use biologically-inspired locomotion control for walking robot including CPG mechanisms (Ijspeert, 2008), decentralized architecture with inter-leg coordination (Cruse et al., 1998; Schilling et al., 2013a) and local leg reflexes (Klaassen et al., 2002; Lewinger and Quinn, 2011). Recently, an increasing number of robots have been designed with a controllable body joint (Boxerbaum et al., 2008; Bartsch et al., 2012; Schilling et al., 2013b).

In contrast to these approaches, our adaptive neural control mechanism combines various key behaviors (i.e., walking, backbone joint behavior, and local leg reflexes) necessary for obstacle negotation. Its neural locomotion control allows the robot to generate robust and energy-efficient walking behavior (Manoonpong et al., 2013b) and a controller for local leg reflexes supports the obstacle negotiation behavior. As the main contribution of this work, the existing concept of an active body joint for hexapod robots (Allen et al., 2003) has been extended by the novel approach of adaptive body joint behavior through learning. Combining these modules, the controller generates adaptive motor behaviors for efficient obstacle negotiation of very high obstacles. Its modularity provides flexible use of the controller. It can be combined with obstacle avoidance behavior triggered by the proximal signal enabling the robot to autonomously learn obstacle negotiation. This behavior is very effective in situations in which the robot is surrounded by obstacles in an unknown and complex environment as shown in Video S1. Furthermore, a laser scanner can be employed for complex task decision making (Kesper et al., 2013). The modular and generic control scheme simplifies transfer from simulated to real robots.

The focus of this work was to coordinate obstacle negotiation behavior of hexapod robots through adaptive and reactive neural control mechanisms. To further increase the climbing capabilities (e.g., increasing climbing speed and climbable obstacle height), this can be achieved by combining the control schemes with mechanisms such as virtual muscle models (Xiong et al., 2013) and biomimetic materials (e.g., shark skin) for the feet and belly (Manoonpong et al., 2013a). In principle, the muscle models can provide compliance and act as simulated mechanical reflexes for minor disturbance rejection and stabilizing the system while the materials can generate frictional anisotropy allowing for firmly grip surfaces during climbing. Furthermore, reservoir computing architectures can be applied for a generic neural control framework increasing temporal signal processing such as short-term memory capacities (Dasgupta et al., 2013). Although the learning mechanism serves as a general framework for obstacle negotiation, learned weights have to be stored by hand for later use. This issue will be resolved by implementing a self-organizing mechanism that stores weights obtained through ICO learning and also preserves the topology of the parameter space given by the obstacle height and the gait parameter.

## Author contributions

Poramate Manoonpong designed research. All authors conceived and designed the experiments, contributed materials and analysis tools and analyzed the data. Dennis Goldschmidt carried out the simulation experiments. Poramate Manoonpong and Dennis Goldschmidt developed the robotic system. Poramate Manoonpong and Dennis Goldschmidt carried out the robotic experiments. Poramate Manoonpong, Dennis Goldschmidt and Florentin Wörgötter worked out the theory. Dennis Goldschmidt and Poramate Manoonpong wrote the manuscript.

## Conflict of interest statement

The authors declare that the research was conducted in the absence of any commercial or financial relationships that could be construed as a potential conflict of interest.

## Supplementary material

Video S1 **Autonomous learning combining adaptive obstacle negotiation with reactive obstacle avoidance**. Simulated AMOS II learns to surmount an 8 cm high obstacle in an autonomous way. An obstacle avoidance reflex, triggered by the proximal signals of the US sensors, causes the robot to turn away from the obstacle. Here the robot learns to negotiate the obstacle after four trials. (http://www.manoonpong.com/AdaptiveClimbing/S1.wmv)

Video S2 **Obstacle negotiation learning for complex obstacles**. We let AMOS II learn to overcome five different complex obstacles: (1) an 8 cm high default obstacle for comparison, (2) a heteromorphic obstacle (i.e., with a 5 cm high left half and an 8 cm right half), (3) an 8 cm high slanted obstacle, (4) an obstacle with the sides declining toward a 5 cm high center box, and (5) stairs with varying step length and 6 cm high steps. The learning rate was set to μ = 0.1 for every obstacle. (http://www.manoonpong.com/AdaptiveClimbing/S2.wmv)

Video S3 **Robust, insect-like negotiation of high obstacles in a real environment**. By applying the weights obtained from ICO learning in the simulation, the robot is able to climb high obstacles in a real environment. Three examples show the climbing capabilities, robustness, and insect-like behavior generated by the controller. The examples correspond to the results shown in Figure 13 of the manuscript. The cockroach videos are taken from (Ritzmann et al., 2004; Lewinger and Quinn, 2009). (http://www.manoonpong.com/AdaptiveClimbing/S3.wmv)
